# Characterization of dairy innovations in selected milksheds in Kenya using a categorical principal component analysis

**DOI:** 10.1007/s11250-021-02596-4

**Published:** 2021-03-25

**Authors:** Edith Wairimu, John Mburu, Charles K. Gachuiri, Asaah Ndambi

**Affiliations:** 1 Department of Agricultural Economics, University of Nairobi, P.O Box 29053, Nairobi, Kenya; 2 Department of Animal Science, University of Nairobi, Nairobi, Kenya; 3 Animal Science Group, Wageningen University and Research, Wageningen, Netherlands

**Keywords:** Cluster, Dairy production, Technical innovations, Organizational innovations, Institutional innovations

## Abstract

To enhance milk quantity and quality which have continued to decrease in Kenya, various stakeholders have intervened through promotion of technical dairy innovations at the farm level including improved cow feeding, health management, promotion of exotic breeds, and milking hygiene. At the milkshed level, stakeholders’ focus has been on organizational innovations, specifically milk sale by farmers through groups. This study sought to characterize dairy innovations that have been adopted by farmers in the milkshed of three milk processors including New Kenya Co-operative Creameries Sotik (NKCC Sotik), Happy Cow Limited (HCL), and Mukurweini Wakulima Dairy Limited (MWDL), representing one state, private, and farmer-owned processor, respectively. Data were collected using a structured questionnaire from a sample of 1146 farmers (410, 382, and 354 in MWDL, HCL, and NKCC Sotik, respectively). A categorical principal components analysis was used to reduce 32 variables into four sets of uncorrelated components. Four categories were identified including principal component (PC) 1 (technical capacity), PC 2 (animal health management), PC 3 (organizational capacity), and PC 4 (milk hygiene). More farmers in the milkshed of MWDL adopted technical and organizational dairy innovations such as use of artificial insemination and milk sale through groups, respectively, than farmers in milkshed of NKCC and HCL. The county governments in the milkshed of HCL and NKCC Sotik need to strengthen cooperative societies to boost adoption of artificial insemination through arrangement in which milk is sold and payment of services offered on credit is settled from milk sale and ensure milk market availability throughout the year.

## Introduction

Dairy production is a key component of the livestock sector in Kenya generating an estimated 14% of the agricultural Gross Domestic Product (GDP) and approximately 4% of Kenya’s total GDP (KDB 2017). As a source of livelihood, the dairy industry supports smallholder dairy farmers summing up to 1.8 million and provides 1.2 million direct and indirect jobs (KDB 2020). In recognition of this significant contribution of the dairy sector to the economy and as a source of livelihoods to smallholder farmers, the national government together with its development partners has been supporting the dairy value chain actors through various public research organizations, universities, training institutes, non-governmental organizations (NGOs), and donor-funded programmes. Their support included development of disease resistant fodder, operationalized strategic milk reserves, and procurement of milk coolers for counties, supporting the dairy hub, on-farm feed production and silage making, dairy infrastructure, ensuring quality based payment system, among others (KDB 2016; Rademaker et al. 2016; Kilelu et al. 2017; Ndambi et al. 2019).

**Fig. 1:**
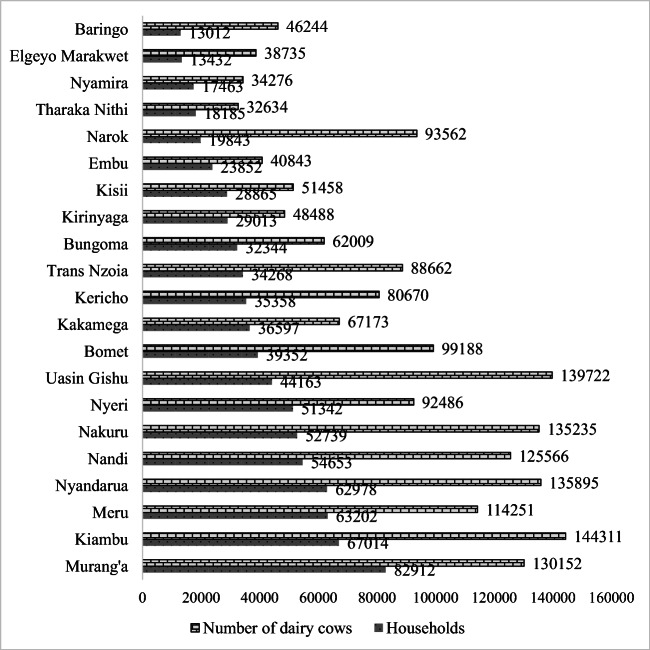
Number of households keeping exotic dairy breeds and number of exotic breeds in 23 out of 47 counties (Source: KNBS 2019)

Although the adoption of promoted dairy innovations remains low (Omondi et al. 2017), there is limited information on already adopted dairy innovations, particularly in the milksheds of Mukurweini Wakulima Dairy Limited (MWDL), Happy Cow Limited (HCL), and NKCC Sotik factory, representing processors that are farmer-owned, privately owned, and state-owned, respectively. A milkshed refers to the milk collection area of a single dairy plant and it can be considered the upstream part of the individual processor’s value chain, from the producers and collectors supplying the processor dairy plant. According to Kenya National Population Census of 2019 (KNBS 2019), the three mentioned milksheds comprise counties that have most of the households keeping exotic breeds in Kenya. For instance, MWDL milkshed comprises three counties Murang’a, Nyeri, and Kirinyaga which account for 8.8%, 5.5%, and 3.1% of the total 939,916 households who keeps exotic dairy breeds in Kenya; HCL comprises Nyandarua, Nakuru, and Baringo county with 6.7%, 5.6%, and 1.4% dairy farms with exotic breeds and NKCC Sotik milkshed hosting counties of Bomet, Kericho, Narok, and Nyamira with 4.2%, 3.8%, 2.1%, and 1.9% farmers keeping exotic dairy breeds (Fig. 1).

Unlike the present study that focused on three categories of dairy innovations (technical, organizational, and institutional), previous studies in these milksheds determined the contribution of one dairy innovation that is aimed at increasing milk production and reducing the effect of seasonality. For example, studies by Richards et al. (2015) and Richards et al. (2019) focused on the effect of feeding high protein fodder trees and other nutritional management factors on the volume of milk sold by smallholder farmers or the impact of feeding minerals on reproductive efficiency on smallholder dairy farms, respectively. Both studies were conducted in the milkshed of MWDL. Another study by Kenduiwa et al. (2016) in Bomet county which is within the milkshed of NKCC Sotik assessed the influence of smallholder dairy farmers’ participation in microfinance on breed improvement, while studies in the milkshed of HCL revealed the importance of smallholder dairy farmer groups in facilitating transformation of new knowledge to action through collaboration between farmers, researchers, and field assistants (Restrepo et al. 2018), and the significance of improved utilization of crop residues such as treating wheat with urea to maintain milk production during the dry season (Kashongwe et al. 2017). In addition, a study by Nyokabi et al. (2018) in the same milkshed revealed that limited enforcement of formal contracts that prescribe the quality of raw milk to be supplied to processors and cooperatives hinders the enhancement of milk quality.

Dairy innovations’ characterization is critical for improving their adoptability, determining potential opportunities and barriers to their adoption, providing platforms for feedback and learning, ensuring the formulation of sector-specific policies, and depicting the production categories that are existing in a particular environment for appropriate introduction of improved technologies (Goswami et al. 2014; Kaouche-Adjlane et al. 2015; Dantas et al. 2016).

The objective of this paper was therefore to characterize dairy innovations adopted by farmers in the year 2018. These included housing of cows, herd management practices, feeding, reproduction, animal health, milk hygiene, milk sale channels, access to credit, and milk sale based on contracts. Since dairy farming is practiced in almost all agro-ecological zones in Kenya, characterizing the adopted dairy innovations is an essential step to provide a practical guideline for the development of appropriate innovation options and policy recommendations.

The remainder of this paper is organized as follows: the concept of dairy innovations and theoretical background of the study, materials and methods, results, discussion, and conclusion and recommendations.

## The concept of innovation in dairy farming and theoretical background of the study

Various authors have defined innovation to include scientific, technological, organizational, financial, and commercial activities needed to produce, implement, and market new or improved products or processes (OECD 1997; Hall et al. 2005). Sumberg (2005) argued that innovation goes beyond science and technology and includes design and institutional innovation. Following these definitions, innovations in the present study included technological, organizational, and institutional. The dairy innovations that are aimed at increased milk quantity and improved milk quality can be classified as technical, organizational, or institutional.

Dairy technical innovations include improved feeding of dairy cows, reproduction management, animal health care, breeding, and housing management with the aim to increase milk production at the farm level. Organizational innovations, on the other hand, include building new milk collection or cooling centers, creating new private collectors or processing companies (or on the contrary setting up direct milk sales to consumers), and creating farmers’ groups or cooperative societies. Institutional innovations include changes in the formal and informal rules shaping the milk collection schemes and milk marketing channels, such as contractual arrangements between farmers and collectors or dairies, milk quality payment schemes, public milk quality regulations, loan programs, or other financial devices. Moreover, institutional innovations also include more informal rules such as consumers’ preferences, consumption habits, and product perception and use.

Collectively, dairy innovations have the potential to improve milk quality, increase milk quantity sold by dairy farmers, and improve the efficiency of the dairy value chain. For instance, technical innovations have been considered likely to increase milk production, decrease seasonality, and improve the microbial quality of milk (Wambugu et al. 2011). To fight exclusion and inefficiency within the milksheds, organizational innovations could stimulate milk production and reduce milk losses (Odero-Waitituh 2017). Similarly, institutional innovations are expected to encourage dairy farmers to improve their production practices (Holloway et al. 2000).

This study was informed by the theory of innovation first proposed by Schumpeter (1934) which explains the role of knowledge and technology in driving productivity and economic growth. The theory explores the various ways such as searching markets, combination of factors of production, sales policy, and innovations in which an entrepreneur can make profits vis-à-vis risk. Additionally, the concept of innovation covers five areas of development that involves new products and services such as production, market, source of raw materials, and organization of industry, all aimed at creating or breaking of a monopoly. These combinations are embodied in unsold raw materials, new technologies, and idle productive capacity. The theory further recognizes credit and finance as key catalysts for innovation. This theory of innovation by Schumpeter (*ibid*) assumed private firms are important in the development of innovations, market is competitive, and financial markets are efficient such that they could support the production of new inventions. The theory specifies the role of innovation in encouraging innovations, enhancing new profitable opportunities and growth in the economy, and improvement in standard of life of the community.

The theory was however only applicable in countries with a democratic system. Over time, authors including Freeman (1982) advanced the Schumpeter theory, emphasized the role of design in innovation, and viewed all economic development as the result of innovation. Whereas Schumpeter (1934) and Freeman (1982) particularly underlined the role of technological innovation, Van de Ven (1993) recognized that the success of technological innovations is determined by institutional innovation representing the social, economic, and political infrastructure required by any community to sustain its members.

Following the argument by Van de Ven (*ibid*), technical dairy innovations are expected to be successful in the presence of developed infrastructure, including organizational innovations. This study is therefore anchored on the theory of simultaneous technical innovations on the farms and organizational/institutional innovations in milksheds in order to increase milk quantity and enhance milk quality.

## Materials and methods

### Study area

The study took place in the milksheds of Mukurweini Wakulima Dairy Limited (MWDL), Happy Cow Limited (HCL), and NKCC Sotik, which are part of the main milksheds in Kenya. The production system in MWDL comprising Nyeri county considered for this study, which is also part of Kenyan highlands is mainly cut and carry system (zero-grazing) (Odero-Waitituh 2017). The HCL milkshed includes three counties: Nakuru, Nyandarua, and Baringo, with the largest proportion of milk being sourced from Nakuru county. This study focused on part of the milkshed within the counties of Nakuru and Nyandarua where up to 70.5% and 16.4% respectively, of HCL milk, was sourced. The majority of farmers practice semi-zero grazing, a system where cows are grazed during the day and are enclosed and offered supplementary feed at night. NKCC Sotik milkshed includes five counties, namely Bomet, Nyamira, Kisii, Narok, and Nakuru. The study was carried out in two counties, Bomet and Nyamira, because up to 80% of milk of NKCC Sotik was sourced from these counties. The production system in this milkshed is mainly a free-range grazing system where cows graze on natural and/or improved pastures using a paddocking or strip grazing approach, and are also supplemented with fodder.

### Sampling procedure and data collection

A multistage sampling technique was used to select farm households for this study. In the first stage, three milksheds from which the three processors (MWDL, HCL, and NKCC Sotik) operate were purposively sampled to represent three processor types: farmer-owned, privately owned, and state-owned, respectively. This selection aimed to clarify if the processor type affected the adoption of innovations by its chain actors. The second stage involved sampling of common milk collection systems across the three milksheds. This stage involved establishment of the criteria for selecting milk collection systems to be considered for the survey. The criteria included systems in which milk is collected and transported to the processor, the possibility of aggregating milk before delivering to the processor, and quality aspects such as initial certification and cooling in cooling plants. Four milk collection systems were finally considered: (i) individual farmers supplying milk directly to the processor (industry), (ii) traders supplying milk to the processor, (iii) cooperative societies where a processor collects milk, and (iv) cooperative societies delivering milk to the processors. The third stage involved purposive selection of the main production areas (sub-locations) in each of the milkshed where the selected milk collection systems are located. An exhaustive list of dairy farmers from all villages within the main production areas (sub-locations) of each milkshed was constituted by respective sub-locations’ administrators while the lists of milk suppliers were collected from processors, cooperative societies/self-help group, and traders. The fourth and the last stage involved a systematic random sampling of dairy farmers. In each of the milk collection systems, a total of 35 suppliers and non-suppliers were targeted. Milk suppliers were farmers delivering milk to the processors through the sampled milk collection systems while non-suppliers were farmers selling their milk to other buyers. To determine the specific respondents to participate in the study, regular intervals were chosen to ensure an adequate sample size. These intervals were determined by dividing the total number of respondents in both lists (milk suppliers and milk non-suppliers) with the target sample of 35. The value that was obtained was then used to determine the specific respondents in the list who were interviewed. To do this, from the two lists of milk suppliers and milk non-suppliers, the starting respondent of the sample was randomly chosen and the interval added to the random number and the process of adding the interval continued until the required sample of 35 was achieved. During data collection from sampled respondents, in the event that the identified respondent was not available, replacement was done in which the immediate respondent in the list was interviewed.

**Fig. 2:**
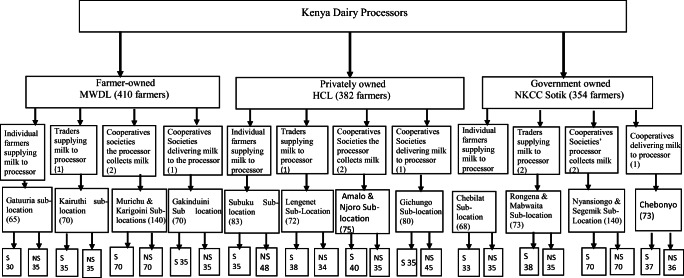
Multistage sampling technique. S means milk suppliers to the processor and NS means milk non-suppliers to the processor

With proportionate to size considerations, a total of 1146 dairy farmers comprising 410, 382, and 354 farmers from the milkshed of MWDL, HCL, and NKCC Sotik, respectively, were sampled. The distribution of the sample by milkshed’s main production areas (sub-locations) is indicated in Fig. 2.

Dairy innovations including cow housing, feeds and feeding, cow reproduction, cow health, milk hygiene, organizational structures, and institutional factors were targeted in the data collection using a structured questionnaire designed in the Open Data Kit (ODK) software. Before actual data collection, the questionnaire was pretested and amended to ensure that all required data was collected for the analysis. Data collection was conducted between July and December 2019 with a reference period of the year 2018. Variables used in the analysis selected from the questionnaire are presented in Table 1.

**Table 1:** Variables used to run categorical principal component analysis

Variable category	Variable name	Variable description and measure
Cow housing	*COWHOUSED*	Whether cows are housed; 1=yes; 0=no
	*FHDRY*	Frequency of housing by season (dry); 1=all the time; 2=night only; 3=occasionally/when need arises (e.g., mating, sick, rain); 4=other specify
	*FHWET*	Frequency of housing by season (wet); 1=all the time; 2=night only; 3=occasionally/when need arises (e.g., mating, sick, rain); 4=other specify
	*MODE_HOUSEDRY*	Mode of cow housing; 1=stable housing 2=other types of housing
	*MODE_HOUSEWET*	Mode of cow housing; 1=stable housing 2=other types of housing
	*LAND_FODDER*	Area under fodder in acres
	*STRFRGE*	Store forage in 2018; 1=yes; 0=no
	*CONCENTFEED*	Feed livestock with concentrates in 2018; 1= yes; 0=no
	*HME_RATIONS*	Home-made rations; 1= yes; 0=no
	*MAINFEEDWET*	The main system of feeding in wet season; 1=only grazing; 2=mainly grazing with some stall feeding;3= mainly stall feeding with some grazing; 4= zero grazing
	*MAINFEEDDRY*	The main system of feeding in dry season; 1=only grazing; 2=manly grazing with some stall feeding; 3= mainly stall feeding with some grazing; 4= zero grazing
Cow Reproduction	*AI*	AI adoption; 1=A1; 2=bull
	*PRP_PURECOWS*	Proportion of pure breeds in the herd; ratio
Cow health	*DEWORMFR*	Frequency of deworming; 1= monthly; 2= 3 months; 3= 6 months
	*DIPSPRAYFR*	Frequency of dipping/spraying; 1= weekly; 2=fortnight; 3=monthly; 4=three months
Milk hygiene	*CLEANTEATS*	Clean teats before milking 1=yes; 0=no
	*PREMIPDT*	Use pre-milking products; 1=yes; 0=no
	*PSTMILK*	Use post milking products; 1=yes; 0=no
	*STMKHR*	Hours milk is stored at home before delivery at collection point
	*HOURSCOOLER*	Hours before milk get to the cooler
	*CLEANEQUIPMENT*	Clean the milking equipment before/after milking; 0=simple water; 2= soap and or disinfectant
	*REFRIGERATE*	Milk refrigerated at home; 1=yes; 0=no
	*DTCTMASTITIS*	Detecting mastitis; 1=yes; 0=no
	*WAITDECISION*	Decision of withdrawal after cows are treated 0= arbitrarily; 1= according to product instructions or veterinary advice
	*WGLOVES*	Wear gloves while milking; 1=yes; 0=no
	*CONTANER_C*	Containers closed during milk storage at home; 1= yes; 0=no
Organizational structures	*MEMBERSHIP*	Group membership; 1=yes; 0=no
	*MILKCOOP*	Selling milk through groups; 1=yes; 0=no
Institutions	*ACC_CREDIT*	Obtained credit; 1=yes; 0=no
	*LONG_TERMLOAN*	Accessed long term loan for dairying; 1=yes; 0=no
	*CONTRCT*	Written agreement (contract) in selling milk; 1=yes; 0=no
	*RECORDS*	keep dairy records; 1=yes; 0=no

### Methods of data analysis

This paper characterized dairy innovations that are adopted by farmers both at the farm and at the milkshed level. The use of CATPCA and cluster analysis in this study was justified by its ability to reveal nonlinear relationships between the variables and jointly analyze numerical, ordinal, and nominal variables through optimal quantification of values of categorical nature to numerical values (Linting et al. 2007; Mair and De Leeuw 2010; Manisera et al. 2010; Linting and Van der Kooij 2012). CATPCA is also useful when two assumptions of PCA including linear relationship between variables and assumption that variables have to be scaled at the numeric level (interval or ratio scale of measurement) are not met. For instance, adoption of dairy innovations may not be linear, given that dairy farmers operate in complex systems where they have to allocate their scarce resources across many enterprises. Contrary to the previous studies that have used other methodologies including principal component analysis and cluster analysis (Martínez-García et al. 2012; Kaouche-Adjlane et al. 2015; Todde et al. 2016; Martin-Collado et al. 2015), factor analysis (Dantas et al. 2016), to characterize dairy farm households based on adopted innovations, socio economic characteristics, and cow traits, this study used CATPCA. To add on, although the study has used similar methodology (CATPCA) like other studies (Abas et al. 2013; Castro et al. 2015; Deng et al. 2019) to characterize dairy innovations at the farm level, this study has used CATPCA analysis to characterize dairy innovations both at the farm and the milkshed level.

### Data analysis

The CATPCA function in SPSS 21, also called non-linear principal component analysis (NLPCA), was used. To exclude highly correlated variables, the Eigen vector plots from PCA were considered. Given that we have measurements on *n* individual on *m* variables given with an *n* × *m* observed score matrix *H* where each variable is denoted by *X*_*j*_*,* j=1…..*m* that is the *j*^*th*^ column of *H*, if the variables *X*_*j*_ are either nominal or ordinal, then optimal scaling is necessary where each observed score is converted into categorical quantification represented by *q* as shown in Equation 1 (Linting et al. 2007).

1 $$ {q}_j=\phi \left({X}_j\right) $$

Furthermore, CATPCA is performed by minimizing the least-squares loss function given in Equation (2) in which the matrix *X* in Equation 1 is replaced by the matrix *Q*.

2 $$ L\left(Q,A,S\right)={n}^{-1}\sum \limits_{i=1}^m tr{\left({q}_j{a}_j^T-S\right)}^T\left({q}_j{a}_j^T-S\right) $$

where *tr* is the trace function, i.e., for any matrix A, the trace function is Equation 3

3 $$ tr\left({A}^TA\right)=\sum \limits_i\sum \limits_j{a}_{ij}^2 $$

The loss function is subjected to some constraints as indicated in Equation 4 with the intention of standardizing the transformed variables to solve interdependence between *q*_*j*_ and *a*_*j*_.

4 $$ {q}_j^T{q}_j=n $$

This standardization indicates that *q*_*j*_ contains *z* scores and produces component loadings in *a*_*j*_ reflecting correlations between the transformed variables and principal components.

The object scores are restricted by Equation 5

5 $$ {S}^TS= nI $$

where *I* is the identity matrix. The object scores are centered as indicated in Equation 6

6 $$ {1}^TS=0 $$

where 1 is a vector of one. The software IBM SPSS Statistics 21 (SPSS, 2012) was used for data analysis. A selection of 32 variables was made from the entire dataset, based on their anticipated ability to capture major variations that described dairy innovations. The selected variables include cow feeds and feeding, cow housing, animal health, reproduction milk hygiene, marketing of milk through cooperative societies, access to credit, and group shareholding. These variables were further reduced to a smaller set of uncorrelated components that represent most of the information found in the original variables (Meulman and Heiser 2012). Using the generated components, a K-means clustering method was then used to characterize farm households with distinctive characteristics by grouping dairy farms that were similar. K-means clustering method was preferred over two-step clustering and hierarchical clustering because the number of clusters was first specified using CATPCA and the data comprised more than 1000 cases Dardac and Boitan (2009).

## Results

Table 2 presents the results of the CATPCA. The analysis yielded four (4) dimensions with Eigen values of 7.18, 2.33, 2.26, and 1.72, respectively. The Cronbach alpha coefficients for the overall model (0.956), as well as for dimensions 1, 2, 3, and 4 (0.889, 0.589, 0.575, and 0.433, respectively), were satisfactory, which means that the test for these samples of farms has a good reliability. Except for one dimension that had a Cronbach alpha of less than 0.500, all the others were near 0.60 which was acceptable. In addition, each item included in the analysis showed a satisfactory loading of more than 0.5 (Hair et al. 2006). The four principal components (PCs) combined explained 42.15% of the total variability in the dataset. The four PCs are characterized by variables with loadings of 0.5 and above (denoted in bold in Table 2). They were named according to these variables.

**Table 2:** Dimensions and component loadings for variables describing dairy innovations (CATPCA results)

Variables*	Dimension			
	1	2	3	4
*COWHOUSED*	**.905**	-.323	-.206	-.127
*FHDRY*	**.905**	-.323	-.206	-.127
*FHWET*	**.905**	-.323	-.206	-.127
MODE_HOUSEDRY	**.914**	-.301	-.206	-.128
MODE_HOUSEWET	**.914**	-.302	-.206	-.128
*MAINFEEDWET*	**.709**	.492	-.144	.180
*MAINFEEDDRY*	**.710**	.491	-.142	.187
*AI*	**−.591**	**−**.248	.048	**−**.271
*DIPSPRAYFR*	.490	**.521**	**−**.249	**−**.012
*MEMBERSHIP*	.405	.391	**.612**	**−**.364
*MILKCOOP*	**−**.405	**−**.391	**−.612**	.364
*CLEANEQUIPMENT*	**−**.134	**−**.180	.119	**.566**
*CONTANER_C*	**−**.102	**−**.042	**−**.005	**.535**
*LAND_FODDER*	.313	**−**.020	.126	.283
*CONCENTFEED*	.419	.082	.073	.333
*STRFRGE*	**−**.060	**−**.380	.139	**−**.085
*HME_RATIONS*	.168	.054	.072	.029
*DEWORMFR*	**−**.022	.107	**−**.162	**−**.025
*CLEANTEATS*	.162	.003	.029	.237
*WGLOVES*	.127	.136	**−**.008	**−**.211
*PREMIPDT*	.352	**−**.185	.406	.088
*PSTMILK*	.319	.294	.180	**−**.029
*HOURSCOOLER*	.313	**−**.101	.498	**−**.022
*STMKHR*	**−**.036	**−**.384	.320	**−**.118
*REFRIGERATE*	.113	**−**.040	.093	.027
*DTCTMASTITIS*	.091	**−**.171	.222	.174
*WAITDECISION*	.255	**−**.272	.241	.286
*ACC_CREDIT*	.388	**−**.142	.411	.137
*LONG_TERMLOAN*	.105	.028	.184	.189
*CONTRCT*	.145	.031	.274	.044
*RECORDS*	.258	**−**.165	.391	.212
*PRP_PURECOWS*	.306	.248	**−**.093	.305
Cronbach’s alpha	.889	.589	.575	.433
Eigen values	7.18	2.33	2.26	1.72
Variance accounted for (%)	22.45	7.28	7.05	5.38

*For abbreviations of variables refer to Table 1

The first PC (technical capacity) explained 22.45% of the total variability in the dataset, with eight variables contributing to this dimension. It was strongly and positively associated with housing of dairy cows at night, and during dry and wet season, zero grazing as main grazing system and use of AI. The second PC (animal health management), which explained additional 7.28% of the total variability, was positively associated with only one variable, namely a low frequency of spraying or dipping cows. Regular spraying and dipping is an essential part of maintaining animal health and consequent milk production and common in semi intensive and extensive production systems to control tick and tick borne diseases is. The third PC (organization capacity) explained an additional 7.05% of total variability and was strongly associated with two variables, including group membership and milk sale through this group. Whereas group membership had a positive correlation with this component, milk sales through the group had a negative effect. Group membership is vital in assuring a steady market for milk year round, reduction of transaction costs (e.g., transport costs, costs for negotiating of contracts, communication costs), and channeling higher investment into dairy farming. The fourth PC (milk hygiene) explained 5.38% of the variability and was positively correlated with cleaning equipment with soap and closing containers while storing milk at home. Milk hygiene is an important component in ensuring milk quality (Table 2).

**Fig. 3:**
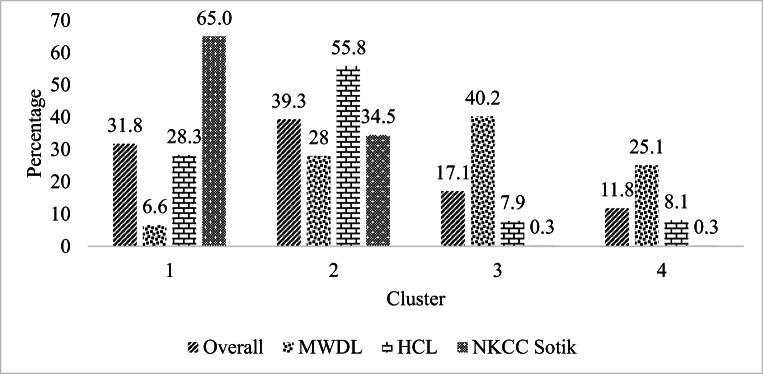
K-means cluster analysis results in percentage by milkshed

To classify the types of dairy innovations in terms of technical, organizational, and institutional across the three milksheds, a K- means clustering technique on principal component was applied. This was done after running CATPCA and a K-means cluster analysis results showed that 365 farms were located in cluster 1, 450 farms in cluster 2, 196 in cluster 3, and 135 farms in cluster 4 (Fig. 3). The clusters were created based on the four dimensions yielded through the CATPCA. Overall, most (39.3%) of respondents were in cluster 2. An analysis across the milksheds indicated that most farmers (65.0% and 55.8%) in milkshed of NKCC Sotik and HCL were in cluster 1 and 2, respectively, while most farmers (40.2%) of MWDL milkshed were in cluster 3 (Fig. 3).

The specific elements identifying the four clusters were determined by examining the percentage analysis of the variables comprising the four PCs describing the main dairy innovations (Table 3). In cluster 1, it was uncommon to house cows at night (95.6%), both during dry and rainy season. The main feeding method during dry and rainy season was only grazing (free-range) or tethering (60.8%). The bull was the main reproduction method used. Cows were sprayed weekly (66.6%) and most of the farmers (76.2%) were not member of a group. Accordingly, only 18.9% sold milk through groups. Use of soap to clean milking equipment and closing of containers while storing milk were common as indicated by 92.1% and 88.0%, respectively.

**Table 3:** Percentage analysis of four dimensions by clusters

	Clusters			
	1	2	3	4
PC 1				
Cows housed at night				
0=no	95.6	0.0	4.1	0.0
1=yes	4.4	100.0	95.9	100.0
Frequency of penning dry				
0=N/A	95.6	0.0	4.1	0.0
1=all the time	3.8	23.8	86.7	96.3
2=night only	0.5	73.3	8.7	3.7
3=occasionally / when need arises (e.g., mating)	0.0	2.9	0.5	0.0
Frequency of penning rainy				
0=N/A	95.6	0.0	4.1	0.0
1=all the time	4.1	24.4	87.8	95.6
2=night only	0.3	72.7	7.7	4.4
3=occasionally / when need arises (e.g., mating)	0.0	2.9	0.5	0.0
Mode housing dry				
0=N/A	95.6	0.0	4.1	0.0
1= open kraal	4.4	36.7	6.1	0.0
2= kraal with roof	0.0	54.9	89.3	0.0
3= brick walled	0.0	0.4	0.0	0.7
5= stable with roof / no pen	0.0	6.2	0.5	94.8
6= in the house	0.0	1.8	0.0	3.7
7= other: (specify in cell)	0.0	0.0	0.0	.7
Mode housing wet				
0=N/A	95.6	0.0	4.1	0.0
1= open kraal	4.4	37.1	5.6	0.0
2= kraal with roof	0.0	54.2	90.3	0.0
3= brick walled	0.0	0.4	0.0	0.7
5= stable with roof / no pen	0.0	6.2	0.0	94.8
6= in the house	0.0	1.8	0.0	3.7
7= other: (specify in cell)	0.0	0.2	0.0	0.7
Main feeding rainy				
1 = only grazing (free-range or tethered)	60.8	26.9	1.5	.7
2 = mainly grazing with some stall feeding	31.2	46.0	6.6	3.0
3 = mainly stall feeding with some grazing	4.9	5.8	4.1	5.9
4 = only stall feeding (zero grazing)	3.0	21.3	87.8	90.4
Main feeding dry				
1 = only grazing (free-range or tethered)	60.8	26.9	1.5	.7
2 = mainly grazing with some stall feeding	31.2	46.0	6.6	3.0
3 = mainly stall feeding with some grazing	4.9	6.0	4.1	5.9
4 = only stall feeding (zero grazing)	3.0	21.1	87.8	90.4
Reproduction method				
1 AI	34.0	67.1	92.9	96.3
2 bull	66.0	32.9	7.1	3.7
PC 2				
Frequency of spraying/dipping				
1= weekly	66.6	61.6	0	13.3
2= fortnight	26.0	31.8	0	13.3
3= monthly	3.8	5.8	20.9	19.3
4= 3 months	1.4	.7	19.4	12.6
5=other	1.4	.2	14.3	4.4
6=none	.8	0.0	45.4	37.0
PC 3				
Group membership				
0=no	76.2	61.1	45.4	42.2
1=yes	23.8	38.9	54.6	57.8
Milk sale through cooperative				
0=no	4.9	5.8	9.7	6.7
1=yes	18.9	33.1	44.9	51.1
2=N/A	76.2	61.1	45.4	42.2
PC 4				
Clean milk equipment				
1 =simple water	5.5	9.1	11.2	38.5
2=soap	92.1	83.6	84.2	55.6
3 =disinfectant		.2	.5	5.2
4 =other (specify)	2.5	7.1	4.1	.7
Close container while storing milk				
0=no	11.5	18.4	8.2	36.3
1=yes	88.5	81.6	91.8	63.7

Cluster 2 presents farms with cow housing at night (100%), with similar proportion during dry season (73.3%) and rainy season (72.7%). The main mode of housing is a kraal with roof (54.2%), and the main mode of feeding is grazing with some stall feeding (46.0% both during dry and rainy seasons). Similar to cluster 1, spraying was done weekly (61.6%). Group membership stands at 38.9%, and 33.1% of respondents sold milk through the group.

In cluster 3, the majority of farmers housed their cows at night, with cow housing taking place at all times during dry and wet season. The main mode of housing is a kraal with roof, both during dry and wet season. Dipping and spraying of cows is uncommon among farmers in cluster 3. The use of AI is more common in cluster 3 compared to clusters 1 and 2. Group membership is high among farmers, and also, milk sale through groups is the highest in this cluster compared to other clusters. Cleaning of milking equipment is mainly done with soap and milk containers are closed while storing milk at home.

Cluster 4 was characterized by farmers who housed their cows at night (100%), all the time during dry and rainy seasons. The main mode of housing during dry and rainy season (94.8%) is a stable with roof and main feeding type during dry and rainy season is stall feeding (zero grazing). Similar to cluster 3, AI is the main reproduction method used by farmers. Group membership and milk sale are common. Cleaning of milking equipment is practiced by slightly above half of respondents (55.6%) while closing of containers storing milk at home was done by 63.7%.

## Discussion

Results of CAPTCA and K-means cluster analysis revealed that the four categories of dairy innovations that were adopted by farmers in the study area differed across the three milksheds. Most (65.0%) farmers of NKCC Sotik (state-owned processor) were in cluster 1 which was characterized by not housing cows at night, use of bull as main method of reproduction, weekly spraying of cows, and low proportion of farmers selling milk through groups. The reason for use of bull in reproduction could be as a result of farmers having large pieces of land to rear bulls (Mwanga et al. 2019), farmers getting discouraged to use AI due to its high cost and cases of repeated inseminations that further increase costs (Mburu et al. 2016). The finding of low use of AI services in the study area was congruent with the findings of Kenduiwa et al. (2016) who studied the influence of smallholder dairy farmers’ participation in microfinance on breed improvement in dairy farming in Bomet County. Farmers could also prefer to graze their cows because they own large pieces of land. Spraying was also done because cows are exposed to tick borne related diseases while under free-range grazing system unlike when animals are in zero-grazing management systems (Omunyin et al. 2014). The low group membership and milk sale through groups, could be associated with low milk production in the milkshed which could be as a result of keeping local breeds and low use of AI services (Kenduiwa et al. 2016).

Regarding privately owned processor’s milkshed (HCL), most (55.8%) of the farmers were classified in cluster 2 which was characterized by housing of cows at night, grazing with some stall feeding, weekly spraying of cows, and low percentage of farmers selling milk through groups. Despite the findings on importance of group membership by a past study by Restrepo et al. (2018) in this milkshed, cooperative membership and milk sale through cooperatives by farmers remained low possibly because of low milk prices offered by groups compared to milk prices by traders. Restrepo et al. (*ibid*) demonstrated that cooperative societies can be used as an avenue for collaborative learning. The study aimed to co-develop local sustainable pathways to reduce milk losses. The results revealed that collaboration between farmers, researchers, and field assistants improved the farmers’ ability to transform their farming system in relation with complex sustainability challenges. The possible reason for low percentage of farmers selling milk through groups could be associated with most farmers selling milk to private milk sellers who offer higher prices than cooperative societies and collect milk at the farmers’ homestead.

In MWDL milkshed (farmer-owned processor), 40.2% of the farmers were in cluster 3 that was commonly associated with housing of cows all the time and zero grazing system, use of AI for reproduction, group membership, and milk sale through groups. Farmers in this milkshed which is part of Central Kenya region mainly practice zero grazing due to their small pieces of land and hence limited land to graze their cows (Bebe et al. 2003). In addition, farmers with small pieces of land may decide to intensify their farming through genetic improvement such as AI (Didanna et al. 2018; Mwanga et al. 2019). In contrast, to the findings of this study, a study on breeding services and factors influencing their use by smallholder dairy farmers in Central Uganda indicated that use of AI was positively influenced by the size of grazing land (Mugisha et al. 2014). Furthermore, small landholdings may explain why farmers adopt a zero grazing system. As a result of zero grazing, cows are not exposed to tick borne diseases as cows under free-range grazing system and hence the cows are not sprayed. The reason for a larger percentage of farmers selling milk through groups unlike in other milksheds could be because farmers in this milkshed keep cow breeds of high genetic potential as represented by 53%, 32%, and 10% of farmers rearing Friesian, Ayrshire, and cross breeds respectively (Nyeri CIDP 2018) and hence producing more milk that is sold through cooperatives. In addition, dairy farmers in this milkshed could be motivated to sell their milk to MWDL (farmer-owned processor) because the processor provides financial services to farmers, including AI and animal health services, livestock feeds, and credit for education fees and feedstuffs which members pay through a check off system (Van Leeuwen et al. 2012). This finding justifies the importance of milk sales through groups and cooperatives in providing an environment suitable for dairy intensification by means of facilitating the dissemination of productivity enhancing technologies and also provides milk marketing services (Chagwiza et al. 2016).

## Conclusion and recommendations

The results of the study revealed that adoption of dairy innovations differed across the milksheds. More farmers in MWDL adopted technical innovations such as AI and organizational innovations including group membership and sale of milk through groups than farmers in the other two milksheds. Based on the findings of this study, there is need to promote the three types of dairy innovations to enhance sustainable milk production quantity and quality. Specifically, the county government in collaboration with other development partners should support farmers particularly in promoting the adoption of AI to improve on genetics. These efforts should target dairy farmers in milksheds of NKCC Sotik and HCL. To promote organizational innovations, farmers in these two milksheds should be supported in forming farmer groups and also offer other services including AI services through a check off system. This is a system in which farmers are offered these services such as AI, feeds, and health services on credit, and the costs is later paid from the milk sale proceeds. Milk sale through groups will help farmers in ensuring milk market all year round. Regarding institutional dairy innovations, the respective county governments and development partners in the three milksheds need to link farmers to financial service providers who can give farmers long term loans to improve their dairying enterprise including purchase of cows of high genetic potential and building houses for cows. Furthermore, dairy farmers should be supported to engage into formal contracts with their buyers which should be based on milk quality and quantity to ensure access to market throughout the year.
